
JOURNAL INFORMATION
==============================
NLM Title Abbreviation: Wirtschaftsdienst
Journal ID: Wirtschaftsdienst
NLM Journal ID: 101086612

Wirtschaftsdienst (Hamburg, Germany : 1949)

ISSN: 0043-6275

ARTICLE INFORMATION
==============================
PMCID: PMC8218288
PMID: 34176989
DOI: 10.1007/s10273-021-2946-x
Article ID: 2946
Article version: 1
Subjects: Ökonomische Trends

Der Einfluss von Mindestlohnerhöhungen auf die Einkommensarmut

The Impact of Minimum Wage Increases on Income Poverty

Kestermann Christian Kestermann@iwkoeln.de
1
Schröder Christoph schroeder.christoph@iwkoeln.de
2
1 grid.473597.b 0000 0000 9854 4639 IW Consult, Institut der deutschen Wirtschaft Köln Consult GmbH, Konrad-Adenauer-Ufer 21, 50668 Köln, Deutschland
2 grid.473597.b 0000 0000 9854 4639 Einkommenspolitik, Arbeitszeiten und -kosten, Institut der deutschen Wirtschaft Köln Consult GmbH, Konrad-Adenauer-Ufer 21, 50668 Köln, Deutschland
Electronic publication date: 2021 Jun 22
Print publication date: 2021
Volume: 101
Issue: 6
First page: 484
Last page: 486
Copyright: © Der/die Autor:in(nen) 2021
License: Open Access: Dieser Artikel wird unter der Creative Commons Namensnennung 4.0 International Lizenz veröffentlicht (https://creativecommons.org/licenses/by/4.0/deed.de).
Open Access wird durch die ZBW — Leibniz-Informationszentrum Wirtschaft gefördert.
License URL: https://creativecommons.org/licenses/by/4.0/

issue-copyright-statement: © The Author(s) 2021

==============================

Im aufkommenden Bundestagswahlkampf rückt das Thema Mindestlohn wieder verstärkt auf die Agenda. Einige Parteien und auch die Gewerkschaften fordern einen Stundenlohn von 12 Euro; „Die Linke“ will sogar einen Anstieg auf 13 Euro. Das Bundesarbeitsministerium und das Finanzministerium haben zu diesem Thema ein Eckpunkte-Papier veröffentlicht, in dem der Anstieg der Lohnuntergrenze auf mindestens 12 Euro im Jahr 2022 gefordert wird – damit würde der geltende Beschluss der Mindestlohnkommission überschrieben (BMAS und BMF, 2021).

Gleichzeitig wird das Ziel formuliert, den Mindestlohn in Richtung eines „Living Wage“ (existenzsichernder Lohn) zu entwickeln und bei zukünftigen Anpassungen den Aspekt der Armutsgefährdung zu berücksichtigen. Als Referenzwert wird 60 % des Medianlohns genannt. Auch in der EU ist das Thema aktuell. Die Kommission hat im Herbst 2020 einen Richtlinienentwurf über „angemessene Mindestlöhne“ vorgelegt. Hierin sind die Mitgliedsländer aufgefordert, Richtwerte im Verhältnis zum allgemeinen Niveau der Bruttolöhne festzusetzen. Empfohlen wird ein Wert von 60 % des Medians oder 50 % des Durchschnitts – also ein Living Wage (Europäische Kommission, 2020, 22).

Bisherige Studien zeigen, dass die Wirkung des Mindestlohns auf die Armutsgefährdung in Deutschland eher gering ist. Untersuchungen von Bruckmeier und Bruttel (2020) mit Daten des Panels Arbeitsmarkt- und Soziale Sicherung (PASS) zeigen zwar einen Rückgang der Armutsgefährdung nach der Mindestlohneinführung. Dieser ist jedoch nur geringfügig größer als in den vorherigen Jahren, sodass kein kausaler Zusammenhang zur Einführung des Mindestlohns hergestellt werden kann. Backhaus und Müller (2019) beobachten auf Basis der Daten des Sozio-oekonomischen Panels (SOEP) ebenfalls keinen signifikanten Rückgang der Armutsgefährdung durch die Mindestlohneinführung. Zudem zeigen sie, dass auch ein Mindestlohn von 12 Euro dies nicht ändern würde. Dieser Aufsatz untersucht die Wirkung eines erhöhten Mindestlohns auf die Einkommensarmut, indem für verschiedene hypothetische Mindestlohnniveaus unter Ceteris-Paribus-Annahmen der Einfluss auf die Armutsgefährdungsquote simuliert wird.

### Beitrag der Mindestlohnempfänger:innen zum Haushaltseinkommen

Unter Verteilungsgesichtspunkten wird ein Mindestlohn am ehesten akzeptiert werden, wenn die Mindestlohnempfänger:innen tatsächlich im besonderen Maß von Einkommensarmut bedroht sind und die Einführung oder Erhöhung des Mindestlohns einen großen Beitrag zur Senkung der Einkommensarmut bei den betroffenen Beschäftigten und ihren Haushaltsangehörigen leisten kann. Tatsächlich ist die Armutsgefährdungsquote der Geringverdienenden jedoch niedrig. So war vor Einführung des Mindestlohns jede/r fünfte Beschäftigte und jede/r siebte Vollzeitbeschäftigte mit geringem Lohn – damals unter 7,50 Euro – von Einkommensarmut betroffen (Heumer et al., 2013). Dies liegt daran, dass die Haushalte sehr oft nicht allein auf die Verdienste der vom Mindestlohn betroffenen Arbeitnehmenden angewiesen sind, sondern ihnen zusätzliche Einkommensquellen durch weitere Haushaltsmitglieder oder andere Einkunftsarten zur Verfügung stehen. Laut Daten des SOEP trugen 2018 drei Viertel aller Beschäftigten mit einem vereinbarten Stundenverdienst zwischen Mindestlohnhöhe und unter 10 Euro mit ihrem Nettoarbeitseinkommen weniger als die Hälfte zum verfügbaren Haushaltseinkommen bei (Schröder und Kestermann, 2020).

Auf Basis der SOEP-Daten für 2018 wird mit einer Simulation der von der Mindestlohnkommission beschlossene und ab Juli 2022 geltende Mindestlohn in Höhe von 10,45 Euro betrachtet. Zudem wird ein Mindestlohn in Höhe des Living Wage – 60 % des Medians des Stundenlohns von Vollzeitarbeitnehmenden – simuliert. Dies sind auf Grundlage der SOEP-Daten aus der 2018er-Erhebung 11,43 Euro und damit 29 % mehr als der damalige Mindestlohn. Der Wert von 10,45 Euro für 2022 muss auf die Verdienstsituation von 2018 übertragen werden. Mit der Umrechnung soll die Abweichung vom regelbasierten Pfad der Mindestlohnerhöhungen beschrieben werden. Regelbasiert folgt der Mindestlohn nachlaufend den Tariflohnsteigerungen der dem Beschlusszeitpunkt vorangegangenen zwei Jahre. Nach dieser Regel wäre der Mindestlohn zum 1. Januar 2021 auf 9,82 Euro erhöht worden und hätte bis Ende 2022 gegolten. Nach dem letzten Mindestlohnbeschluss steigt der Mindestlohn jedoch bis Juli 2022 in mehreren Stufen auf 10,45 Euro und liegt dann 6,4 % über der regelbasierten Anpassung. Da der gesetzliche Mindestlohn im Jahr 2018 bei 8,84 Euro lag, entsprechen die 10,45 Euro für das Jahr 2022 im Jahr 2018 somit 9,40 Euro.

Tabelle 1
**Simulation eines höheren Mindestlohns**
Veränderung der Armutsgefährdungsquote gegenüber den Ist-Werten in Prozentpunkten

| Simulierter Mindestlohn | Beschäftigte | Bevölkerung |
|---|---|---|
| Status quo Euro ohne Unterschreiter | -0,5 | -0,5 |
| Anhebung auf 10,45 Euro 2022 mit Unterschreitern | -0,5 | -0,2 |
| Anhebung auf 10,45 Euro 2022 ohne Unterschreiter | -0,9 | -0,6 |
| Living Wage mit Unterschreitern | -1,3 | -0,6 |
| Living Wage ohne Unterschreiter | -1,4 | -1,0 |

Anmerkungen: Basis: Äquivalenzeinkommen (modifizierte OECD-Skala) auf Grundlage des 2018 detailliert abgefragten Vorjahreseinkommens (einschließlich des Mietwerts selbstgenutzter Eigentümerwohnungen); ohne Unterschreiter: der (berechnete) vereinbarte Stundenlohn wird für Unterschreiter auf den gesetzlichen Mindestlohn (Status quo) bzw. den simulierten Mindestlohn angehoben; mit Unterschreitern: Für Beschäftigte, die weniger als den im Status quo gültigen Mindestlohn verdienen, werden die Stundenverdienste proportional zur simulierten Anhebung des Mindestlohns erhöht.

## Simulation höherer Mindestlöhne

Für die beiden hypothetischen Mindestlöhne werden je zwei Varianten simuliert:

- *Variante 1*: Alle Verdienste zwischen 8,84 Euro und dem jeweiligen hypothetischen Mindestlohn werden auf den neuen Wert erhöht. Verdienste unter 8,84 Euro werden proportional zur hypothetischen Erhöhung des Mindestlohns angehoben. Hinter dieser Überlegung steckt, dass es vermutlich auch bei einer Anhebung des Mindestlohns Unterschreiter geben wird, die nicht den Mindestlohn erhalten.

- *Variante 2*: Bei dieser Variante werden im Vergleich zur Variante 1 auch die Stundenverdienste unter 8,84 Euro auf den jeweiligen hypothetischen Mindestlohn angehoben. Es wird also unterstellt, dass es keine Unterschreitungen mehr gibt.

Als zusätzliche Alternative wird die vollständige Einhaltung des damaligen Mindestlohns von 8,84 Euro simuliert.

Ausgehend von den erhöhten Stundenlöhnen wurde die Erhöhung des Bruttomonatslohns mit den im SOEP angegebenen (vertraglich vereinbarten) Arbeitszeiten errechnet, womit unterstellt wird, dass es keine Arbeitszeitreduzierungen der Betriebe als Reaktion auf die Lohnerhöhungen gibt. Von dieser Bruttoerhöhung wurden wiederum pauschal 30 % für Steuern und Sozialversicherungsbeiträge abgezogen, um näherungsweise die Nettolohnerhöhung zu ermitteln. Schließlich wurden die Haushaltseinkommen um die Nettolohnanstiege aller betroffenen Haushaltsmitglieder erhöht und der Schwellenwert für Einkommensarmut entsprechend angepasst.

Es zeigt sich, dass bei allen Simulationen der Rückgang bei der Einkommensarmutsquote sowohl mit Blick auf die Beschäftigten als auch auf die Gesamtbevölkerung maximal 1,4 Prozentpunkte beträgt (vgl. Tabelle 1). Die Wirkung der simulierten Erhöhung ist bei den Beschäftigten tendenziell größer als in der Gesamtbevölkerung. Beispielsweise verringert ein simulierter Mindestlohn in Höhe von 10,45 Euro mit Unterschreitern die Armutsgefährdungsquote der Beschäftigten um 0,5 Prozentpunkte, die der Gesamtbevölkerung dagegen lediglich um 0,2 Prozentpunkte. Bei einem simulierten Mindestlohn auf Höhe des Living Wage ist die Wirkung auf die Armutsgefährdungsquote unter den Beschäftigten ebenfalls etwa doppelt so hoch wie in der Gesamtbevölkerung, wenn es bei der jetzigen Zahl und Intensität der Unterschreitungen bleibt.

In den Varianten ohne Unterschreitung sind die Unterschiede zwischen den betrachteten Mindestlohnhöhen ähnlich hoch wie bei den Varianten mit Unterschreitern. Die Simulation eines Mindestlohns von 10,45 Euro im Jahr 2022 ohne Unterschreitung bewirkt eine Senkung der Armutsgefährdungsquote um 0,6 Prozentpunkte bei der Gesamtbevölkerung und um 0,9 Prozentpunkte unter den Beschäftigten. Ein fiktiver Mindestlohn in Höhe des Living Wage verringert die Armutsgefährdungsquote in der Gesamtbevölkerung um 1 Prozentpunkt und bei den Beschäftigten um 1,4 Prozentpunkte.

Vergleicht man gleiche Szenarien – also jeweils mit oder ohne Unterschreiter – brächte die Einführung des Living Wage in der Gesamtbevölkerung gegenüber der bereits beschlossenen Anhebung auf 10,45 Euro lediglich eine Absenkung der Armutsgefährdungsquote von 0,4 Prozentpunkten. Auch unter den Beschäftigten würde der Living-Wage den Anteil der Einkommensarmen im Vergleich zur 10,45-Euro-Simulation nur um 0,5 oder 0,8 Prozentpunkte senken.

Ebenfalls rund einen halben Prozentpunkt betragen die Unterschiede in der Reduktion der Armutsgefährdungsquote bei allen Mindestlohnhöhen zwischen der Variante mit Unterschreitung und der Variante mit strikter Einhaltung des Mindestlohns. Dies gilt sowohl für die Beschäftigten als auch für die Gesamtbevölkerung. Die einzige Ausnahme ergibt sich für die Armutsgefährdung der Beschäftigten bei der Einführung eines Living Wage als Lohnuntergrenze – hier beträgt der Unterschied zwischen den beiden Varianten nur 0,1 Prozentpunkte. Daher lässt sich das Armutsrisiko in der Gesamtbevölkerung durch einen Living Wage mit Unterschreitungen nicht wesentlich stärker senken als durch eine strikte Einhaltung des bisher vereinbarten Mindestlohns.

Die Simulation läuft insbesondere unter zwei Ceteris-Paribus-Annahmen: Zum einen wird angenommen, dass es keine Beschäftigungseffekte gibt. Verschiedene Studien zeigen jedoch negative Effekte auf die Beschäftigung und die vereinbarte Arbeitszeit bei einer starken Erhöhung des Mindestlohns, wie bereits bei der Mindestlohneinführung zu sehen war (Ahlfeldt et al., 2019; Knabe et al., 2020). Damit würde die Armutsgefährdungsquote vermutlich weniger stark sinken als simuliert. Bei einem Rückgang der vereinbarten Arbeitszeit steigen die Monatslöhne schwächer als die Stundenlöhne, sodass sich die positiven Effekte auf die Armutsgefährdungsquote abschwächen. Kommt es zu einem deutlichen Anstieg der Arbeitslosigkeit, könnten die Effekte eines zu hohen Mindestlohns sogar kontraproduktiv sein.

Auf der anderen Seite gibt es mögliche positive Sekundäreffekte durch die simulierte Lohnerhöhung. Die Regelsätze der Grundsicherung und die Renten könnten etwas stärker steigen, wenn die Lohnerhöhungen auch zu Preiserhöhungen führen und damit Anpassungen der Arbeitslosengeld-II-Leistungen nach sich ziehen. Zudem führt die Anhebung des Mindestlohns insgesamt zu einem Lohndruck, der insbesondere die Entgelte im unteren Entgeltbereich steigen lassen könnte, wie es bereits nach Einführung des Mindestlohns zu sehen war (Schröder und Kestermann, 2020).

Die Simulationsergebnisse zeigen, dass die Erhöhung des Mindestlohns auch im Idealfall keiner negativen Auswirkungen auf die Beschäftigung nur einen geringen Effekt auf die Armutsgefährdungsquote in Deutschland hätte. Daher ist sie nicht als geeignete Maßnahme zu sehen, um Erwerbseinkommensarmut und die Armutsgefährdungsquote in der Gesamtbevölkerung zielgenau zu senken. Insbesondere deuten unsere Ergebnisse daraufhin, dass eine reine Erhöhung wenig Wirkung zeigt, wenn gleichzeitig die Zahl der Beschäftigten, die weniger als den Mindestlohn verdienen, konstant bleibt.

## Mindestlohn muss bezahlbar bleiben

Wichtig ist es daher, den Mindestlohn so festzusetzen, dass er für die Unternehmen bezahlbar bleibt und die Arbeitnehmer:innen Qualifizierungsanreize erhalten, damit sie nicht dauerhaft auf den Mindestlohn angewiesen sind. Eine zu starke Stauchung der Löhne im unteren Bereich der Verdienstverteilung könnte hier kontraproduktiv wirken. Bereits die beschlossene Erhöhung auf 10,45 Euro im Juli 2022 ist angesichts der COVID-19-Pandemie und der damit verbunden wirtschaftlichen Schwächung gerade der Kleinbetriebe im Dienstleistungsgewerbe eine große Herausforderung.

Christian Kestermann ist Referent bei der IW Consult am Institut der deutschen Wirtschaft (IW) in Köln.

Christoph Schröder ist Senior Researcher am Institut der deutschen Wirtschaft (IW) in Köln.


Literatur

Ahlfeldt, G., D. Roth und T. Seidel (2019), Employment-maximizing minimum wages, Preliminary Version, https://www.aeaweb.org/conference/2020/preliminary/paper/3yk65y8G (11. Mai 2021).
BMAS (Bundesministerium für Arbeit und Soziales) und BMF (Bundesministerium der Finanzen) (2021), Fairer Mindestlohn — Starke Sozialpartnerschaft, https://www.bmas.de/SharedDocs/Downloads/DE/Arbeitsrecht/fairer-mindestlohn.html#direct_download (11. Mai 2021).
Backhaus, T. und K.-U. Müller (2019), Does the German Minimum Wage Help Low Income Households? Evidence from Observed Outcomes and the Simulation of Potential Effects, Discussion Papers, 1805, https://www.diw.de/documents/publikationen/73/diw_01.c.623948.de/dp1805.pdf (11. Mai 2021).
Bruckmeier K Bruttel O Minimum Wage as a Social Policy Instrument: Evidence from Germany Journal of Social Policy 2020 50 2 1 20
Europäische Kommission (2020), Vorschlag für eine Richtlinie des Europäischen Parlaments und des Rates über angemessene Mindestlöhne in der Europäischen Union vom 28.10.2020, COM(2020) 682 final, https://ec.europa.eu/transparency/regdoc/rep/1/2020/DE/COM-2020-682-F1-DE-MAIN-PART-1.PDF (11. Mai 2021).
Heumer M Lesch H Schröder C Mindestlohn, Einkommensverteilung und Armutsrisiko IW-Trends 2013 40 1 19 36
Knabe A Schöb R Thum M Prognosen und empirische Befunde: Wie groß ist die Kluft beim Mindestlohn wirklich? Perspektiven der Wirtschaftspolitik 2020 21 1 25 29 10.1515/pwp-2020-0008
Schröder C Kestermann C Mindestlohn und Einkommensarmut IW-Trends 2020 47 2 107 127
